# Supplementary material for: Targeted Muscle Reinnervation (TMR) or Regenerative Peripheral Nerve Interface (RPNI) for pain prevention in patients with limb amputation: A protocol for a systematic review and meta-analysis
Source: PLoS One. 2026 Jun 5;21(6):e0350845. doi: 10.1371/journal.pone.0350845 (PMC13240872; doi:10.1371/journal.pone.0350845)
Supplement: S1 File — Full electronic search strategy, including keywords and boolean operators, for Cochrane Central Register of Controlled Trials (CENTRAL), Web of Science, Scopus, PubMed/MEDLINE, CINAHL and Google Scholar. (DOCX) [file pone.0350845.s001.docx]

# Search strategy

Búsquedas realizadas el 2-06-2025

**PubMed**

((((((((((Limb amputation[Title/Abstract]) OR (Extremity amputation[Title/Abstract])) OR (Amputation[Title/Abstract])) OR (Amputee[Title/Abstract])) OR (Amputate[Title/Abstract])) OR (Limb disarticulation[Title/Abstract])) OR (Extremity disarticulation[Title/Abstract])) OR (Disarticulation[Title/Abstract])) OR (Amputation, Surgical[MeSH Terms])) OR (Disarticulation[MeSH Terms])) AND ((((((((Targeted muscle reinnervation[Title/Abstract]) OR (Targeted muscle re-innervation[Title/Abstract])) OR (TMR[Title/Abstract])) OR (Regenerative peripheral nerve interface[Title/Abstract])) OR (RPNI[Title/Abstract])) OR (nerve transfer[Title/Abstract])) OR (TMRPNI[Title/Abstract])) OR (Nerve transfer[MeSH Terms]))

[330 results](https://pubmed.ncbi.nlm.nih.gov/?term=%28%28%28%28%28%28%28%28%28%28Limb+amputation%5BTitle%2FAbstract%5D%29+OR+%28Extremity+amputation%5BTitle%2FAbstract%5D%29%29+OR+%28Amputation%5BTitle%2FAbstract%5D%29%29+OR+%28Amputee%5BTitle%2FAbstract%5D%29%29+OR+%28Amputate%5BTitle%2FAbstract%5D%29%29+OR+%28Limb+disarticulation%5BTitle%2FAbstract%5D%29%29+OR+%28Extremity+disarticulation%5BTitle%2FAbstract%5D%29%29+OR+%28Disarticulation%5BTitle%2FAbstract%5D%29%29+OR+%28Amputation%2C+Surgical%5BMeSH+Terms%5D%29%29+OR+%28Disarticulation%5BMeSH+Terms%5D%29%29+AND+%28%28%28%28%28%28%28%28Targeted+muscle+reinnervation%5BTitle%2FAbstract%5D%29+OR+%28Targeted+muscle+re-innervation%5BTitle%2FAbstract%5D%29%29+OR+%28TMR%5BTitle%2FAbstract%5D%29%29+OR+%28Regenerative+peripheral+nerve+interface%5BTitle%2FAbstract%5D%29%29+OR+%28RPNI%5BTitle%2FAbstract%5D%29%29+OR+%28nerve+transfer%5BTitle%2FAbstract%5D%29%29+OR+%28TMRPNI%5BTitle%2FAbstract%5D%29%29+OR+%28Nerve+transfer%5BMeSH+Terms%5D%29%29&sort=relevance&size=200)

**WOS**

(((((((((((TI="Limb amputation" OR AB="Limb amputation")) OR ((TI="Extremity amputation" OR AB="Extremity amputation"))) OR ((TI=Amputation OR AB=Amputation))) OR ((TI=Amputee OR AB=Amputee))) OR ((TI=Amputate OR AB=Amputate))) OR ((TI="Limb disarticulation" OR AB="Limb disarticulation"))) OR ((TI="Extremity disarticulation" OR AB="Extremity disarticulation"))) OR ((TI=Disarticulation OR AB=Disarticulation))) OR (ALL="Amputation, Surgical")) OR (ALL=Disarticulation)) AND (((((((((TI="Targeted muscle reinnervation" OR AB="Targeted muscle reinnervation")) OR ((TI="Targeted muscle re-innervation" OR AB="Targeted muscle re-innervation"))) OR ((TI=TMR OR AB=TMR))) OR ((TI="Regenerative peripheral nerve interface" OR AB="Regenerative peripheral nerve interface"))) OR ((TI=RPNI OR AB=RPNI))) OR ((TI="nerve transfer" OR AB="nerve transfer"))) OR ((TI=TMRPNI OR AB=TMRPNI))) OR (ALL="Nerve transfer"))

[347 results](https://www-webofscience-com.ezproxy.unav.es/wos/woscc/summary/3592c75d-e316-45b4-a6f8-4785beb11f67-0165f226f1/relevance/1)

**Scopus**

((((((((((TITLE-ABS("Limb amputation")) OR (TITLE-ABS("Extremity amputation"))) OR (TITLE-ABS(Amputation))) OR (TITLE-ABS(Amputee))) OR (TITLE-ABS(Amputate))) OR (TITLE-ABS("Limb disarticulation"))) OR (TITLE-ABS("Extremity disarticulation"))) OR (TITLE-ABS(Disarticulation))) OR (INDEXTERMS("Amputation, Surgical"))) OR (INDEXTERMS(Disarticulation))) AND ((((((((TITLE-ABS("Targeted muscle reinnervation")) OR (TITLE-ABS("Targeted muscle re-innervation"))) OR (TITLE-ABS(TMR))) OR (TITLE-ABS("Regenerative peripheral nerve interface"))) OR (TITLE-ABS(RPNI))) OR (TITLE-ABS("nerve transfer"))) OR (TITLE-ABS(TMRPNI))) OR (INDEXTERMS("Nerve transfer")))

[443 results](https://www-scopus-com.ezproxy.unav.es/results/results.uri?sort=plf-f&src=s&sid=c598a097e688ce35758bf5a6f0e3af46&sot=a&sdt=a&sl=652&s=%28%28%28%28%28%28%28%28%28%28TITLE-ABS%28%22Limb+amputation%22%29%29+OR+%28TITLE-ABS%28%22Extremity+amputation%22%29%29%29+OR+%28TITLE-ABS%28Amputation%29%29%29+OR+%28TITLE-ABS%28Amputee%29%29%29+OR+%28TITLE-ABS%28Amputate%29%29%29+OR+%28TITLE-ABS%28%22Limb+disarticulation%22%29%29%29+OR+%28TITLE-ABS%28%22Extremity+disarticulation%22%29%29%29+OR+%28TITLE-ABS%28Disarticulation%29%29%29+OR+%28INDEXTERMS%28%22Amputation%2C+Surgical%22%29%29%29+OR+%28INDEXTERMS%28Disarticulation%29%29%29+AND+%28%28%28%28%28%28%28%28TITLE-ABS%28%22Targeted+muscle+reinnervation%22%29%29+OR+%28TITLE-ABS%28%22Targeted+muscle+re-innervation%22%29%29%29+OR+%28TITLE-ABS%28TMR%29%29%29+OR+%28TITLE-ABS%28%22Regenerative+peripheral+nerve+interface%22%29%29%29+OR+%28TITLE-ABS%28RPNI%29%29%29+OR+%28TITLE-ABS%28%22nerve+transfer%22%29%29%29+OR+%28TITLE-ABS%28TMRPNI%29%29%29+OR+%28INDEXTERMS%28%22Nerve+transfer%22%29%29%29&origin=searchadvanced&editSaveSearch=&txGid=5f14f0ff90c9aee60bba0cbe691c03c2&sessionSearchId=c598a097e688ce35758bf5a6f0e3af46&limit=10)

**Cochrane**

(((((((((("Limb amputation":ti,ab) OR ("Extremity amputation":ti,ab)) OR (Amputation:ti,ab)) OR (Amputee:ti,ab)) OR (Amputate:ti,ab)) OR ("Limb disarticulation":ti,ab)) OR ("Extremity disarticulation":ti,ab)) OR (Disarticulation:ti,ab)) OR ([mh "Amputation, Surgical"])) OR ([mh Disarticulation])) AND (((((((("Targeted muscle reinnervation":ti,ab) OR ("Targeted muscle re-innervation":ti,ab)) OR (TMR:ti,ab)) OR ("Regenerative peripheral nerve interface":ti,ab)) OR (RPNI:ti,ab)) OR ("nerve transfer":ti,ab)) OR (TMRPNI:ti,ab)) OR ([mh "Nerve transfer"]))

[17 trials](https://www.cochranelibrary.com/advanced-search/search-manager)

**CINAHL**

(((((((((((TI "Limb amputation" OR AB "Limb amputation")) OR ((TI "Extremity amputation" OR AB "Extremity amputation"))) OR ((TI Amputation OR AB Amputation))) OR ((TI Amputee OR AB Amputee))) OR ((TI Amputate OR AB Amputate))) OR ((TI "Limb disarticulation" OR AB "Limb disarticulation"))) OR ((TI "Extremity disarticulation" OR AB "Extremity disarticulation"))) OR ((TI Disarticulation OR AB Disarticulation))) OR ((MH "Amputation, Surgical+"))) OR ((MH Disarticulation+))) AND (((((((((TI "Targeted muscle reinnervation" OR AB "Targeted muscle reinnervation")) OR ((TI "Targeted muscle re-innervation" OR AB "Targeted muscle re-innervation"))) OR ((TI TMR OR AB TMR))) OR ((TI "Regenerative peripheral nerve interface" OR AB "Regenerative peripheral nerve interface"))) OR ((TI RPNI OR AB RPNI))) OR ((TI "nerve transfer" OR AB "nerve transfer"))) OR ((TI TMRPNI OR AB TMRPNI))) OR ((MH "Nerve transfer+")))

[56 results](https://web-p-ebscohost-com.ezproxy.unav.es/ehost/resultsadvanced?sid=c7df4ff7-1a39-4df6-be27-bf1c97a269a6%40redis&vid=17&bk=1&bquery=(((((((((((TI+%22Limb+amputation%22+OR+AB+%22Limb+amputation%22))+OR+((TI+%22Extremity+amputation%22+OR+AB+%22Extremity+amputation%22)))+OR+((TI+Amputation+OR+AB+Amputation)))+OR+((TI+Amputee+OR+AB+Amputee)))+OR+((TI+Amputate+OR+AB+Amputate)))+OR+((TI+%22Limb+disarticulation%22+OR+AB+%22Limb+disarticulation%22)))+OR+((TI+%22Extremity+disarticulation%22+OR+AB+%22Extremity+disarticulation%22)))+OR+((TI+Disarticulation+OR+AB+Disarticulation)))+OR+((MH+%22Amputation%2c+Surgical%2b%22)))+OR+((MH+Disarticulation%2b)))+AND+(((((((((TI+%22Targeted+muscle+reinnervation%22+OR+AB+%22Targeted+muscle+reinnervation%22))+OR+((TI+%22Targeted+muscle+re-innervation%22+OR+AB+%22Targeted+muscle+re-innervation%22)))+OR+((TI+TMR+OR+AB+TMR)))+OR+((TI+%22Regenerative+peripheral+nerve+interface%22+OR+AB+%22Regenerative+peripheral+nerve+interface%22)))+OR+((TI+RPNI+OR+AB+RPNI)))+OR+((TI+%22nerve+transfer%22+OR+AB+%22nerve+transfer%22)))+OR+((TI+TMRPNI+OR+AB+TMRPNI)))+OR+((MH+%22Nerve+transfer%2b%22)))&bdata=JmRiPWM4aCZsYW5nPWVzJnR5cGU9MSZzZWFyY2hNb2RlPVN0YW5kYXJkJnNpdGU9ZWhvc3QtbGl2ZSZzY29wZT1zaXRl)

**Google scholar**

(amputation OR disarticulation OR amputee) AND (targeted muscle reinnervation OR TMR OR Regenerative peripheral nerve interface OR RPNI OR TMRPNI)

[6230 Results.](https://scholar.google.es/scholar?hl=es&as_sdt=0%2C5&q=%28amputation+OR+disarticulation+OR+amputee%29+AND+%28targeted+muscle+reinnervation+OR+TMR+OR+Regenerative+peripheral+nerve+interface+OR+RPNI+OR+TMRPNI%29&btnG=) Se cogen los primeros 500 resultados
