# Supplementary material for: Targeted Muscle Reinnervation (TMR) or Regenerative Peripheral Nerve Interface (RPNI) for pain prevention in patients with limb amputation: A protocol for a systematic review and meta-analysis
Source: PLoS One. 2026 Jun 5;21(6):e0350845. doi: 10.1371/journal.pone.0350845 (PMC13240872; doi:10.1371/journal.pone.0350845)
Supplement: Attachment — Submitted filename: Cover Letter _ Response to Reviewers.docx [file pone.0350845.s003.docx]

Cover Letter / Response to Reviewers

**Date:** 9/01/2026

**Manuscript Number:** PONE-D-25-31923

**Title:** Targeted Muscle Reinnervation (TMR) or Regenerative Peripheral Nerve Interface (RPNI) for pain prevention in patients with limb amputation: a protocol for a systematic review and meta-analysis

**To:** Simone Ranaldi, Ph.D. Academic Editor PLOS ONE

Dear Dr. Ranaldi,

Thank you for giving us the opportunity to submit a revised version of our manuscript. We greatly appreciate the time and effort you and the reviewers dedicated to providing such insightful and constructive feedback. We have carefully reviewed all comments and have revised the manuscript accordingly. Furthermore, we believe these changes have significantly strengthened the protocol, improving its clarity, methodological rigor, and readability.

Please find below a point-by-point response to the reviewers’ comments, detailing the specific changes made.

Regarding the file formats: Due to the extensive nature of the revisions requested, we have rewritten large sections of the manuscript to improve flow and structure. Consequently, the "Revised Manuscript with Track Changes" file displays modifications to nearly the entire text, which may make it difficult to read. To facilitate the review process, we kindly suggest referring primarily to the "Clean Version" of the manuscript alongside our detailed response below.

### **Response to Reviewer #1**

**General Comment:** *“Does the manuscript provide a valid rationale...? Yes. Is the protocol technically sound...? Yes.”*

**Response:** We thank the reviewer for their positive assessment of our rationale and technical soundness.

**1. Method clarity: Define “classical amputation” more precisely.**

**Response:** We agree that this term can be ambiguous. We have added a precise definition in the Methods section. We now explicitly state that "classical amputation" refers to standard amputation techniques (myoplasty or myodesy) performed without any additional prophylactic nerve handling procedures such as TMR or RPNI.

**2. Data synthesis: Clarify standardization for different pain scales.**

**Response:** We have updated the *Data Synthesis* section to clarify that when studies use different continuous scales to measure the same outcome (e.g., VAS 0-10 vs. PROMIS measures), we will use the **Standardized Mean Difference (SMD)** rather than the Mean Difference (MD) to pool the results in the meta-analysis. This ensures statistical comparability.

**3. Limitations: Add a dedicated limitations section.**

**Response:** We have added a dedicated "Strengths and Limitations" section to the manuscript (previously part of the discussion/intro) to explicitly discuss potential limitations, such as the expected heterogeneity of the included studies and the potential inclusion of case series.

**4. Figures: Include a PRISMA-P diagram.**

**Response:** We have included the PRISMA-P checklist and ensuring the flow of the protocol aligns with PRISMA-P guidelines. A PRISMA flow diagram will be generated once the review is completed (results stage), but we have referenced the PRISMA-P checklist in the supplementary files as requested.

**5. Supplementary: Include the full search strategy as a supplement.**

**Response:** We apologize if this file was not accessible in the previous submission. We have ensured that the full search strategy (listing keywords and syntax for all databases) is correctly uploaded and labeled as *S1 File*.

**6. Language: Minor editorial corrections for grammar and clarity.**

**Response:** We have conducted a comprehensive linguistic revision of the entire manuscript. In addition to addressing specific grammatical errors, we have extensively edited the text to enhance its clarity, flow, and readability, consistent with the feedback provided by Reviewer #2.

### **Response to Reviewer #2**

**General Comment:** *“The core work of the study can be original and worthwhile publishing provided that the authors address the points below.”*

**Response:** We sincerely thank the reviewer for seeing the potential in our work and for the detailed critique which has helped us refine the protocol significantly.

**Critique on Style**

We sincerely appreciate the reviewer’s observation regarding the writing style and the need for professional editing. We have taken this feedback very seriously. Furthermore, we have conducted a comprehensive linguistic revision and rewriting of the entire manuscript to improve clarity, flow, and academic tone. Consequently, the Introduction and Methods sections have been substantially rephrased to meet the journal's standards.

This extensive revision addresses the specific stylistic points raised:

- **L 49 (Point 5):** Terminology has been standardized to “upper or lower limb amputation” throughout the text.
- **L 24, 71, 82, 90, 95 (Points 2, 8, 10, 11, 13):** These sentences have been completely rewritten or integrated into new paragraphs to ensure grammatical correctness and better readability.
- **L 266 (Point 21):** Spelling errors (e.g., IPC) have been corrected or eliminated.

**Critique on References (Old references) & Documentation:**

**Response:** We agree with this observation. We have updated the bibliography to prioritize recent literature (2020–2025), particularly in the Introduction regarding amputation epidemiology and pain management, to ensure the clinical context is up to date. Additionally, we have verified that all supplementary materials (Search Strategy, Data Extraction Form) are correctly attached and accessible.

**Response to Specific Technical Comments:**

**1. L 25: Consider adding the lower time limit for the reference selection, if any.**

**Response:** We have clarified in the Methods that there are no lower time limits for study inclusion to ensure comprehensive coverage. Anyway, we expect most relevant TMR/RPNI studies to be recent.

**3. L 38: Verify if RPNI and TMR are acknowledged MeSH terms.**

**Response:** We have verified this. While TMR and RPNI are not yet specific MeSH headings, we agree that spelling them out improves discoverability. We have updated the Keywords section to include the full terms "Targeted Muscle Reinnervation" and "Regenerative Peripheral Nerve Interface".

**4. L 43: Modify this statement about “high-quality articles”…**

**Response:** We agree that the original phrasing was contradictory given our inclusion criteria. We have revised the “Strengths and Limitations” section to state: *“-Through a rigorous selection process compliant with PRISMA standards, this review will collate the totality of the available evidence to evaluate the safety and efficacy of RPNI and TMR, assessing the certainty of findings using GRADE.”*

**6. L 54: Add “Classical amputation without reinnervation”.**

**Response:** Added for clarity.

**7. L 56 & L 200: Categorize outcomes according to measure of efficacy and safety.**

**Response:** We have reorganized the *Outcomes* section. We now explicitly categorize them under “Efficacy Outcomes” (Neuroma, PLP, RLP, Pain scores) and “Safety Outcomes” (Surgical complications).

**9. L 77-80: Add some statistical data to support these points.**

**Response:** We have extensively revised this section in the Introduction. We now explicitly cite recent projections estimating over 3.6 million individuals living with limb loss by 2050, as well as specific data on demographic disparities, such as the four-fold higher risk of major amputation in Black patients [Refs 1-4].

**12. L 92: Table 1 should include the spelling of US and MRI.**

**Response:** To improve the flow of the manuscript, we have integrated the information regarding diagnostic criteria for neuromas directly into the Methods section. We have clarified that we will extract data based on study definitions, noting when standardized criteria like Arnold's are used. Within that text, we have ensured that the abbreviations are fully spelled out as “Ultrasound” and “Magnetic Resonance Imaging”.

**14. L 111: Include information about other treatments for PLP.**

**Response:** We have briefly mentioned other treatments (e.g., mirror therapy, medication) to provide context on why surgical prophylaxis is distinct/important.

**15. L 123: Provide references looking at one or the other or both interventions.**

**Response:** We have ensured that all statements regarding the individual efficacy of TMR and RPNI are now supported by specific, recent references. In the Introduction, we explicitly cite Zimbulis et al. [12] for TMR efficacy, Hooper et al. [13] and Kubiak et al. [14] for RPNI outcomes, and De Lange et al. [15] and Mauch et al. [16] as systematic reviews covering both interventions.

**16. L 165: Included other critical confounders.**

**Response:** Excellent suggestion. We have added “Surgical team specialty”, “adjuvant oncological treatment (neurotoxic chemotherapy or radiotherapy)” and “pre-operative pain status (to account for central sensitization)” to our list of data to be extracted and considered in subgroup analyses or meta-regression, as these are critical effect modifiers.

**17. L 200: Indicate clearly to outcomes related to efficacy and safety.**

**Response:** Please refer to our response to Comment #7. We have applied this categorization throughout the manuscript, clearly distinguishing between Efficacy and Safety outcomes in the Methods section.

**18. L 212 & 20 (L 239): Provide list of keywords and data extraction form.**

**Response:** The complete Search Strategy (keywords and syntax) has been uploaded as Supplementary Material (S1 File). Regarding the Data Extraction Form, we utilize Covidence software for the review process; therefore, the form is a digital template integrated within the platform rather than a standalone document. However, to ensure full transparency and comply with your request, we have explicitly listed every variable and field used in this digital template within the "Data Extraction and Management" section of the manuscript.

**19. L 226: Reference the Table for timeline.**

**Response:** To streamline the manuscript, we have replaced the timeline table with a narrative description. The project schedule and updated completion dates (2026) are now explicitly detailed in the text of the Methods section (under the subheading *Project Timeline*).

**21. L 266: Spell IPC in caption of Table**

**Response:** The table containing this abbreviation has been removed during the manuscript revision to streamline the text. The relevant information is now presented in narrative form in the Methods section, ensuring no undefined abbreviations remain.

**22 & 23 (L 314-315): Heading for “References” and older references.**

**Response:** We have added the “References” heading. Furthermore, we have conducted a major update of the bibliography, replacing older statistics with literature from 2015-2025 to ensure the protocol reflects the current state of the art.

__________

We hope that the revised manuscript now meets the high standards of PLOS ONE. We look forward to your decision.

Sincerely,

**David Gurpegui**

Department of Plastic, Aesthetic and Reconstructive Surgery

Clínica Universidad de Navarra

Pamplona, Spain

dgurpegui@unav.es

Appendix

# Reporting checklist for protocol of a systematic review and meta analysis.

Based on the PRISMA-P guidelines.

## **Instructions to authors**

Complete this checklist by entering the page numbers from your manuscript where readers will find each of the items listed below.

Your article may not currently address all the items on the checklist. Please modify your text to include the missing information. If you are certain that an item does not apply, please write "n/a" and provide a short explanation.

Upload your completed checklist as an extra file when you submit to a journal.

In your methods section, say that you used the PRISMA-Preporting guidelines, and cite them as:

Moher D, Shamseer L, Clarke M, Ghersi D, Liberati A, Petticrew M, Shekelle P, Stewart LA. Preferred Reporting Items for Systematic Review and Meta-Analysis Protocols (PRISMA-P) 2015 statement. Syst Rev. 2015;4(1):1.

|  |  | Reporting Item | Page Number |
| --- | --- | --- | --- |
| **Title** |  |  |  |
| Identification | [#1a](https://www.goodreports.org/reporting-checklists/prisma-p/info/#1a) | Identify the report as a protocol of a systematic review | 1 |
| Update | [#1b](https://www.goodreports.org/reporting-checklists/prisma-p/info/#1b) | If the protocol is for an update of a previous systematic review, identify as such | n/a |
| **Registration** |  |  |  |
|  | [#2](https://www.goodreports.org/reporting-checklists/prisma-p/info/#2) | If registered, provide the name of the registry (such as PROSPERO) and registration number | 2 |
| **Authors** |  |  |  |
| Contact | [#3a](https://www.goodreports.org/reporting-checklists/prisma-p/info/#3a) | Provide name, institutional affiliation, e-mail address of all protocol authors; provide physical mailing address of corresponding author | 1 |
| Contribution | [#3b](https://www.goodreports.org/reporting-checklists/prisma-p/info/#3b) | Describe contributions of protocol authors and identify the guarantor of the review | 14 |
| **Amendments** |  |  |  |
|  | [#4](https://www.goodreports.org/reporting-checklists/prisma-p/info/#4) | If the protocol represents an amendment of a previously completed or published protocol, identify as such and list changes; otherwise, state plan for documenting important protocol amendments | n/a |
| **Support** |  |  |  |
| Sources | [#5a](https://www.goodreports.org/reporting-checklists/prisma-p/info/#5a) | Indicate sources of financial or other support for the review | 14 |
| Sponsor | [#5b](https://www.goodreports.org/reporting-checklists/prisma-p/info/#5b) | Provide name for the review funder and / or sponsor | n/a |
| Role of sponsor or funder | [#5c](https://www.goodreports.org/reporting-checklists/prisma-p/info/#5c) | Describe roles of funder(s), sponsor(s), and / or institution(s), if any, in developing the protocol | n/a |
| **Introduction** |  |  |  |
| Rationale | [#6](https://www.goodreports.org/reporting-checklists/prisma-p/info/#6) | Describe the rationale for the review in the context of what is already known | 3-5 |
| Objectives | [#7](https://www.goodreports.org/reporting-checklists/prisma-p/info/#7) | Provide an explicit statement of the question(s) the review will address with reference to participants, interventions, comparators, and outcomes (PICO) | 5 |
| **Methods** |  |  |  |
| Eligibility criteria | [#8](https://www.goodreports.org/reporting-checklists/prisma-p/info/#8) | Specify the study characteristics (such as PICO, study design, setting, time frame) and report characteristics (such as years considered, language, publication status) to be used as criteria for eligibility for the review | 6-7 |
| Information sources | [#9](https://www.goodreports.org/reporting-checklists/prisma-p/info/#9) | Describe all intended information sources (such as electronic databases, contact with study authors, trial registers or other grey literature sources) with planned dates of coverage | 8 |
| Search strategy | [#10](https://www.goodreports.org/reporting-checklists/prisma-p/info/#10) | Present draft of search strategy to be used for at least one electronic database, including planned limits, such that it could be repeated | S1 |
| Study records - data management | [#11a](https://www.goodreports.org/reporting-checklists/prisma-p/info/#11a) | Describe the mechanism(s) that will be used to manage records and data throughout the review | 8-9 |
| Study records - selection process | [#11b](https://www.goodreports.org/reporting-checklists/prisma-p/info/#11b) | State the process that will be used for selecting studies (such as two independent reviewers) through each phase of the review (that is, screening, eligibility and inclusion in meta-analysis) | 8-9 |
| Study records - data collection process | [#11c](https://www.goodreports.org/reporting-checklists/prisma-p/info/#11c) | Describe planned method of extracting data from reports (such as piloting forms, done independently, in duplicate), any processes for obtaining and confirming data from investigators | 8-9 |
| Data items | [#12](https://www.goodreports.org/reporting-checklists/prisma-p/info/#12) | List and define all variables for which data will be sought (such as PICO items, funding sources), any pre-planned data assumptions and simplifications | 9 |
| Outcomes and prioritization | [#13](https://www.goodreports.org/reporting-checklists/prisma-p/info/#13) | List and define all outcomes for which data will be sought, including prioritization of main and additional outcomes, with rationale | 9 |
| Risk of bias in individual studies | [#14](https://www.goodreports.org/reporting-checklists/prisma-p/info/#14) | Describe anticipated methods for assessing risk of bias of individual studies, including whether this will be done at the outcome or study level, or both; state how this information will be used in data synthesis | 10 |
| Data synthesis | [#15a](https://www.goodreports.org/reporting-checklists/prisma-p/info/#15a) | Describe criteria under which study data will be quantitatively synthesised | 10 |
| Data synthesis | [#15b](https://www.goodreports.org/reporting-checklists/prisma-p/info/#15b) | If data are appropriate for quantitative synthesis, describe planned summary measures, methods of handling data and methods of combining data from studies, including any planned exploration of consistency (such as I2, Kendall’s τ) | 10 |
| Data synthesis | [#15c](https://www.goodreports.org/reporting-checklists/prisma-p/info/#15c) | Describe any proposed additional analyses (such as sensitivity or subgroup analyses, meta-regression) | 11-12 |
| Data synthesis | [#15d](https://www.goodreports.org/reporting-checklists/prisma-p/info/#15d) | If quantitative synthesis is not appropriate, describe the type of summary planned | n/a |
| Meta-bias(es) | [#16](https://www.goodreports.org/reporting-checklists/prisma-p/info/#16) | Specify any planned assessment of meta-bias(es) (such as publication bias across studies, selective reporting within studies) | 11 |
| Confidence in cumulative evidence | [#17](https://www.goodreports.org/reporting-checklists/prisma-p/info/#17) | Describe how the strength of the body of evidence will be assessed (such as GRADE) | 12 |

The PRISMA-P elaboration and explanation paper is distributed under the terms of the Creative Commons Attribution License CC-BY. This checklist was completed on 30. November 2024 using <https://www.goodreports.org/>, a tool made by the [EQUATOR Network](https://www.equator-network.org) in collaboration with [Penelope.ai](https://www.penelope.ai)

# **Search strategy**

Búsquedas realizadas el 2-06-2025

**PubMed**

((((((((((Limb amputation[Title/Abstract]) OR (Extremity amputation[Title/Abstract])) OR (Amputation[Title/Abstract])) OR (Amputee[Title/Abstract])) OR (Amputate[Title/Abstract])) OR (Limb disarticulation[Title/Abstract])) OR (Extremity disarticulation[Title/Abstract])) OR (Disarticulation[Title/Abstract])) OR (Amputation, Surgical[MeSH Terms])) OR (Disarticulation[MeSH Terms])) AND ((((((((Targeted muscle reinnervation[Title/Abstract]) OR (Targeted muscle re-innervation[Title/Abstract])) OR (TMR[Title/Abstract])) OR (Regenerative peripheral nerve interface[Title/Abstract])) OR (RPNI[Title/Abstract])) OR (nerve transfer[Title/Abstract])) OR (TMRPNI[Title/Abstract])) OR (Nerve transfer[MeSH Terms]))

[330 results](https://pubmed.ncbi.nlm.nih.gov/?term=%28%28%28%28%28%28%28%28%28%28Limb+amputation%5BTitle%2FAbstract%5D%29+OR+%28Extremity+amputation%5BTitle%2FAbstract%5D%29%29+OR+%28Amputation%5BTitle%2FAbstract%5D%29%29+OR+%28Amputee%5BTitle%2FAbstract%5D%29%29+OR+%28Amputate%5BTitle%2FAbstract%5D%29%29+OR+%28Limb+disarticulation%5BTitle%2FAbstract%5D%29%29+OR+%28Extremity+disarticulation%5BTitle%2FAbstract%5D%29%29+OR+%28Disarticulation%5BTitle%2FAbstract%5D%29%29+OR+%28Amputation%2C+Surgical%5BMeSH+Terms%5D%29%29+OR+%28Disarticulation%5BMeSH+Terms%5D%29%29+AND+%28%28%28%28%28%28%28%28Targeted+muscle+reinnervation%5BTitle%2FAbstract%5D%29+OR+%28Targeted+muscle+re-innervation%5BTitle%2FAbstract%5D%29%29+OR+%28TMR%5BTitle%2FAbstract%5D%29%29+OR+%28Regenerative+peripheral+nerve+interface%5BTitle%2FAbstract%5D%29%29+OR+%28RPNI%5BTitle%2FAbstract%5D%29%29+OR+%28nerve+transfer%5BTitle%2FAbstract%5D%29%29+OR+%28TMRPNI%5BTitle%2FAbstract%5D%29%29+OR+%28Nerve+transfer%5BMeSH+Terms%5D%29%29&sort=relevance&size=200)

**WOS**

(((((((((((TI="Limb amputation" OR AB="Limb amputation")) OR ((TI="Extremity amputation" OR AB="Extremity amputation"))) OR ((TI=Amputation OR AB=Amputation))) OR ((TI=Amputee OR AB=Amputee))) OR ((TI=Amputate OR AB=Amputate))) OR ((TI="Limb disarticulation" OR AB="Limb disarticulation"))) OR ((TI="Extremity disarticulation" OR AB="Extremity disarticulation"))) OR ((TI=Disarticulation OR AB=Disarticulation))) OR (ALL="Amputation, Surgical")) OR (ALL=Disarticulation)) AND (((((((((TI="Targeted muscle reinnervation" OR AB="Targeted muscle reinnervation")) OR ((TI="Targeted muscle re-innervation" OR AB="Targeted muscle re-innervation"))) OR ((TI=TMR OR AB=TMR))) OR ((TI="Regenerative peripheral nerve interface" OR AB="Regenerative peripheral nerve interface"))) OR ((TI=RPNI OR AB=RPNI))) OR ((TI="nerve transfer" OR AB="nerve transfer"))) OR ((TI=TMRPNI OR AB=TMRPNI))) OR (ALL="Nerve transfer"))

[347 results](https://www-webofscience-com.ezproxy.unav.es/wos/woscc/summary/3592c75d-e316-45b4-a6f8-4785beb11f67-0165f226f1/relevance/1)

**Scopus**

((((((((((TITLE-ABS("Limb amputation")) OR (TITLE-ABS("Extremity amputation"))) OR (TITLE-ABS(Amputation))) OR (TITLE-ABS(Amputee))) OR (TITLE-ABS(Amputate))) OR (TITLE-ABS("Limb disarticulation"))) OR (TITLE-ABS("Extremity disarticulation"))) OR (TITLE-ABS(Disarticulation))) OR (INDEXTERMS("Amputation, Surgical"))) OR (INDEXTERMS(Disarticulation))) AND ((((((((TITLE-ABS("Targeted muscle reinnervation")) OR (TITLE-ABS("Targeted muscle re-innervation"))) OR (TITLE-ABS(TMR))) OR (TITLE-ABS("Regenerative peripheral nerve interface"))) OR (TITLE-ABS(RPNI))) OR (TITLE-ABS("nerve transfer"))) OR (TITLE-ABS(TMRPNI))) OR (INDEXTERMS("Nerve transfer")))

[443 results](https://www-scopus-com.ezproxy.unav.es/results/results.uri?sort=plf-f&src=s&sid=c598a097e688ce35758bf5a6f0e3af46&sot=a&sdt=a&sl=652&s=%28%28%28%28%28%28%28%28%28%28TITLE-ABS%28%22Limb+amputation%22%29%29+OR+%28TITLE-ABS%28%22Extremity+amputation%22%29%29%29+OR+%28TITLE-ABS%28Amputation%29%29%29+OR+%28TITLE-ABS%28Amputee%29%29%29+OR+%28TITLE-ABS%28Amputate%29%29%29+OR+%28TITLE-ABS%28%22Limb+disarticulation%22%29%29%29+OR+%28TITLE-ABS%28%22Extremity+disarticulation%22%29%29%29+OR+%28TITLE-ABS%28Disarticulation%29%29%29+OR+%28INDEXTERMS%28%22Amputation%2C+Surgical%22%29%29%29+OR+%28INDEXTERMS%28Disarticulation%29%29%29+AND+%28%28%28%28%28%28%28%28TITLE-ABS%28%22Targeted+muscle+reinnervation%22%29%29+OR+%28TITLE-ABS%28%22Targeted+muscle+re-innervation%22%29%29%29+OR+%28TITLE-ABS%28TMR%29%29%29+OR+%28TITLE-ABS%28%22Regenerative+peripheral+nerve+interface%22%29%29%29+OR+%28TITLE-ABS%28RPNI%29%29%29+OR+%28TITLE-ABS%28%22nerve+transfer%22%29%29%29+OR+%28TITLE-ABS%28TMRPNI%29%29%29+OR+%28INDEXTERMS%28%22Nerve+transfer%22%29%29%29&origin=searchadvanced&editSaveSearch=&txGid=5f14f0ff90c9aee60bba0cbe691c03c2&sessionSearchId=c598a097e688ce35758bf5a6f0e3af46&limit=10)

**Cochrane**

(((((((((("Limb amputation":ti,ab) OR ("Extremity amputation":ti,ab)) OR (Amputation:ti,ab)) OR (Amputee:ti,ab)) OR (Amputate:ti,ab)) OR ("Limb disarticulation":ti,ab)) OR ("Extremity disarticulation":ti,ab)) OR (Disarticulation:ti,ab)) OR ([mh "Amputation, Surgical"])) OR ([mh Disarticulation])) AND (((((((("Targeted muscle reinnervation":ti,ab) OR ("Targeted muscle re-innervation":ti,ab)) OR (TMR:ti,ab)) OR ("Regenerative peripheral nerve interface":ti,ab)) OR (RPNI:ti,ab)) OR ("nerve transfer":ti,ab)) OR (TMRPNI:ti,ab)) OR ([mh "Nerve transfer"]))

[17 trials](https://www.cochranelibrary.com/advanced-search/search-manager)

**CINAHL**

(((((((((((TI "Limb amputation" OR AB "Limb amputation")) OR ((TI "Extremity amputation" OR AB "Extremity amputation"))) OR ((TI Amputation OR AB Amputation))) OR ((TI Amputee OR AB Amputee))) OR ((TI Amputate OR AB Amputate))) OR ((TI "Limb disarticulation" OR AB "Limb disarticulation"))) OR ((TI "Extremity disarticulation" OR AB "Extremity disarticulation"))) OR ((TI Disarticulation OR AB Disarticulation))) OR ((MH "Amputation, Surgical+"))) OR ((MH Disarticulation+))) AND (((((((((TI "Targeted muscle reinnervation" OR AB "Targeted muscle reinnervation")) OR ((TI "Targeted muscle re-innervation" OR AB "Targeted muscle re-innervation"))) OR ((TI TMR OR AB TMR))) OR ((TI "Regenerative peripheral nerve interface" OR AB "Regenerative peripheral nerve interface"))) OR ((TI RPNI OR AB RPNI))) OR ((TI "nerve transfer" OR AB "nerve transfer"))) OR ((TI TMRPNI OR AB TMRPNI))) OR ((MH "Nerve transfer+")))

[56 results](https://web-p-ebscohost-com.ezproxy.unav.es/ehost/resultsadvanced?sid=c7df4ff7-1a39-4df6-be27-bf1c97a269a6%40redis&vid=17&bk=1&bquery=(((((((((((TI+%22Limb+amputation%22+OR+AB+%22Limb+amputation%22))+OR+((TI+%22Extremity+amputation%22+OR+AB+%22Extremity+amputation%22)))+OR+((TI+Amputation+OR+AB+Amputation)))+OR+((TI+Amputee+OR+AB+Amputee)))+OR+((TI+Amputate+OR+AB+Amputate)))+OR+((TI+%22Limb+disarticulation%22+OR+AB+%22Limb+disarticulation%22)))+OR+((TI+%22Extremity+disarticulation%22+OR+AB+%22Extremity+disarticulation%22)))+OR+((TI+Disarticulation+OR+AB+Disarticulation)))+OR+((MH+%22Amputation%2c+Surgical%2b%22)))+OR+((MH+Disarticulation%2b)))+AND+(((((((((TI+%22Targeted+muscle+reinnervation%22+OR+AB+%22Targeted+muscle+reinnervation%22))+OR+((TI+%22Targeted+muscle+re-innervation%22+OR+AB+%22Targeted+muscle+re-innervation%22)))+OR+((TI+TMR+OR+AB+TMR)))+OR+((TI+%22Regenerative+peripheral+nerve+interface%22+OR+AB+%22Regenerative+peripheral+nerve+interface%22)))+OR+((TI+RPNI+OR+AB+RPNI)))+OR+((TI+%22nerve+transfer%22+OR+AB+%22nerve+transfer%22)))+OR+((TI+TMRPNI+OR+AB+TMRPNI)))+OR+((MH+%22Nerve+transfer%2b%22)))&bdata=JmRiPWM4aCZsYW5nPWVzJnR5cGU9MSZzZWFyY2hNb2RlPVN0YW5kYXJkJnNpdGU9ZWhvc3QtbGl2ZSZzY29wZT1zaXRl)

**Google scholar**

(amputation OR disarticulation OR amputee) AND (targeted muscle reinnervation OR TMR OR Regenerative peripheral nerve interface OR RPNI OR TMRPNI)

[6230 Results.](https://scholar.google.es/scholar?hl=es&as_sdt=0%2C5&q=%28amputation+OR+disarticulation+OR+amputee%29+AND+%28targeted+muscle+reinnervation+OR+TMR+OR+Regenerative+peripheral+nerve+interface+OR+RPNI+OR+TMRPNI%29&btnG=) Se cogen los primeros 500 resultados.
